# Machine learning reveals that *Mycobacterium tuberculosis* genotypes and anatomic disease site impacts drug resistance and disease transmission among patients with proven extra-pulmonary tuberculosis

**DOI:** 10.1186/s12879-020-05256-4

**Published:** 2020-07-31

**Authors:** Doctor B. Sibandze, Beki T. Magazi, Lesibana A. Malinga, Nontuthuko E. Maningi, Bong-Akee Shey, Jotam G. Pasipanodya, Nontombi N. Mbelle

**Affiliations:** 1 Department of Medical Microbiology, Faculty of Health Sciences, University of Pretoria, Private Bag X323, Arcadia, Pretoria, 0007 South Africa; 2 National Tuberculosis Control Program, Ministry of Health, Manzini, Kingdom of Eswatini; 3 Tshwane Division, National Health Laboratory Services, Pretoria, South Africa; 4 Tuberculosis Platform, South African Medical Research Council, Pretoria, South Africa; 5 Center For Infectious Diseases Research and Experimental Therapeutics, Texas Tech University Health Sciences Center, 5920 Forest Park Road, Dallas, TX 75235 USA; 6 Praedicare Laboratories, 14830 Venture Drive, Dallas, TX 75234 USA

**Keywords:** Stochastic gradient boosting, Spoligotypes, Number needed to screen, Attributable risk, Pharmacokinetic variability, Acquired drug resistance

## Abstract

**Background:**

There is a general dearth of information on extrapulmonary tuberculosis (EPTB). Here, we investigated *Mycobacterium tuberculosis (*Mtb*)* drug resistance and transmission patterns in EPTB patients treated in the Tshwane metropolitan area, in South Africa.

**Methods:**

Consecutive Mtb culture-positive non-pulmonary samples from unique EPTB patients underwent mycobacterial genotyping and were assigned to phylogenetic lineages and transmission clusters based on spoligotypes. MTBDR*plus* assay was used to search mutations for isoniazid and rifampin resistance. Machine learning algorithms were used to identify clinically meaningful patterns in data. We computed odds ratio (OR), attributable risk (AR) and corresponding 95% confidence intervals (CI).

**Results:**

Of the 70 isolates examined, the largest cluster comprised 25 (36%) Mtb strains that belonged to the East Asian lineage. East Asian lineage was significantly more likely to occur within chains of transmission when compared to the Euro-American and East-African Indian lineages: OR = 10.11 (95% CI: 1.56–116). Lymphadenitis, meningitis and cutaneous TB, were significantly more likely to be associated with drug resistance: OR = 12.69 (95% CI: 1.82–141.60) and AR = 0.25 (95% CI: 0.06–0.43) when compared with other EPTB sites, which suggests that poor rifampin penetration might be a contributing factor.

**Conclusions:**

The majority of Mtb strains circulating in the Tshwane metropolis belongs to East Asian, Euro-American and East-African Indian lineages. Each of these are likely to be clustered, suggesting on-going EPTB transmission. Since 25% of the drug resistance was attributable to sanctuary EPTB sites notorious for poor rifampin penetration, we hypothesize that poor anti-tuberculosis drug dosing might have a role in the development of resistance.

## Background

South Africa has one of the highest tuberculosis and human immunodeficiency virus (TB/HIV) incidence rate per capita, with the World Health Organization (WHO) estimating new case incidences of 834 per 100, 000 population in 2015 and 520 in 2019 [1]. Recently, WHO set stringent tuberculosis (TB) elimination milestones and targets for member countries. The milestones are 10 new TB cases per million people per year by the year 2035, while the target goal is 1 case per million people per year by the year 2050. Nonetheless, it is estimated that 15 to 20% of all TB notified cases might have disease restricted to extra-pulmonary sites (EPTB), such as meningeal, lymphatic, cutaneous or pericardial space. However, the true proportion of proven TB at such anatomical sites is not well described [2, 3]. In order to meet the WHO TB elimination targets South Africa will need to undertake more vigorous TB surveillance and direct more resources towards EPTB efforts. However, there are still misinformed beliefs among some public health practitioners and TB programs that EPTB, including childhood TB, do not constitute public health threats because EPTB is less likely to be transmitted between persons. It is for these and other reasons that childhood TB was not reportable and therefore not formally captured in public records by many national TB programs until 2012 [4]. The net effect has been to regard all TB lesions, except for those from the bronchus, lung parenchyma and bronchopulmonary lymph nodes, in one obscure category called EPTB and devote even fewer resources to the disease [5–7]. As a result, *Mycobacterium tuberculosis* (Mtb) genotypes, drug resistance patterns and temporal trends associated with EPTB are not well described in South Africa, or in the Tshwane Municipality [3].

**Fig. 1:**
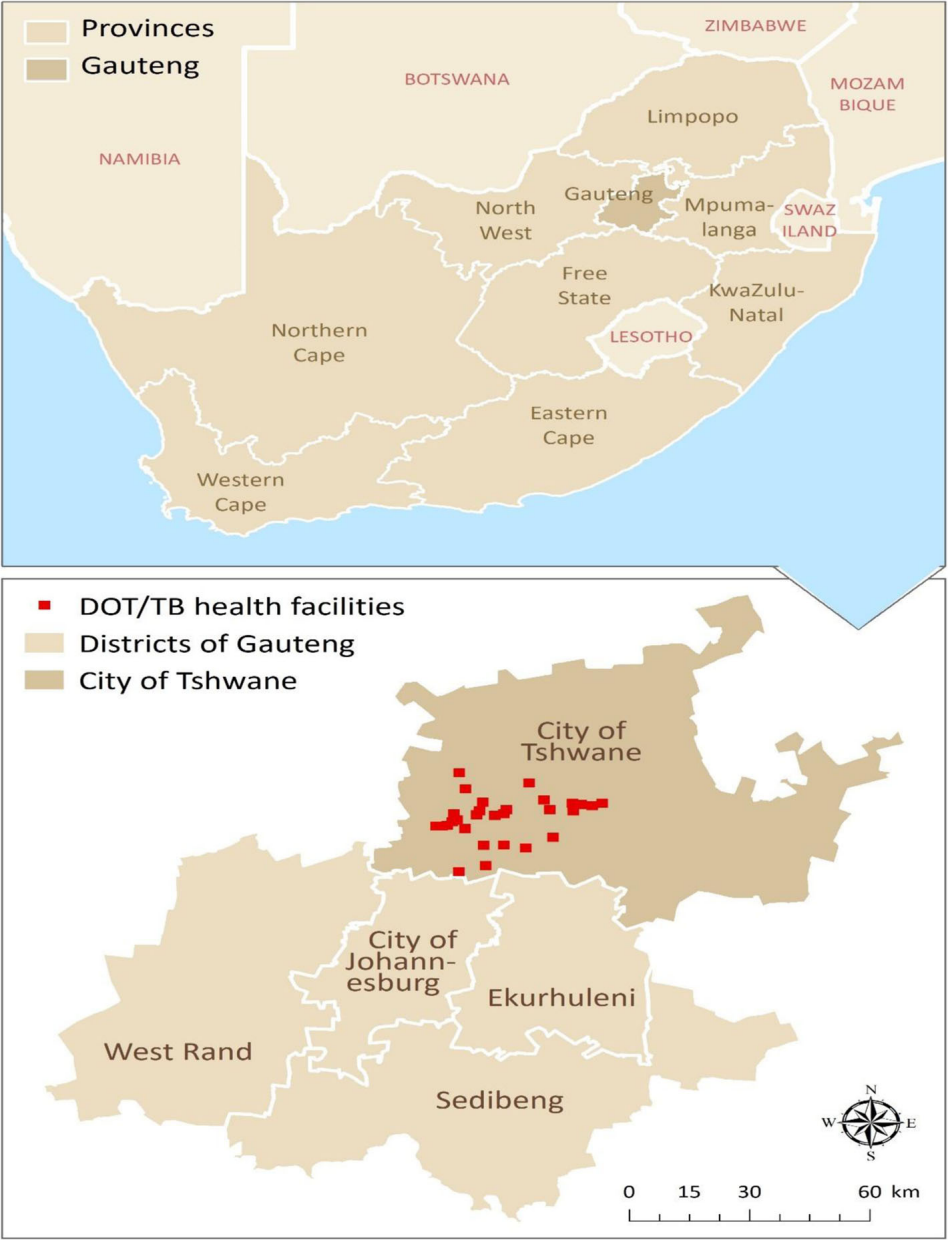
Geographic location of tuberculosis (TB) services and directly observed treatment strategy (DOTS) centers in Tshwane municipality of South Africa

The city of Tshwane, in Gauteng province, is the financial hub and most densely populated municipality in South Africa. The Tshwane Metropolitan area covers a region of 6368 km^2^ and is supported by a sophisticated network of > 25 directly observed treatment strategy (DOTS)/TB centres and tertiary level teaching facilities that serve a multi-ethnic and diverse population in excess of 3 million people, including migrants from across sub-Saharan Africa (Fig. 1). This makes Tshwane an ideal place to obtain generalizable findings on transmission dynamics of different Mtb genotypes with or without drug resistance within diverse populations. The predominant strains associated with the pulmonary (PTB) epidemic in the Gauteng province of South Africa are the globally prevalent, modern and re-imported Lineages 4, particularly the Latin-American Mediterranean (LAM) sublineages and the East Asian Lineage 2, which has the moniker “Beijing” strains [2, 8–11]. Both lineages affect geographically diverse human populations worldwide, have been associated with rapid human-to-human transmission and hence greater propensity for acquired drug resistance [9, 12, 13]. On the other hand, the same cannot be said about the more ancient East African Indian Lineage 3, such as EAI1_SOM families, which have also been isolated with equal frequency in PTB patients from Gauteng province [8, 10, 14]. Here, we wanted to identify factors that predict drug resistance in EPTB patients in Tshwane. In order to inform policy, we specifically wanted to examine the interaction between drug resistance and TB transmission among EPTB patients in the Tshwane metropolis.

There are several molecular tools that promote the rapid identification of drug resistance patterns of Mtb complex directly from clinical specimen and allow the analysis of molecular clock within and against the reference [2, 15]. These genotyping technologies can assign Mtb isolates into distinct clusters or groups based on their relatedness with respect to time (phylogeny), geography and other characteristics, in order to ascertain and compare disease transmission between groups. Mtb genotyping is relatively simple, readily available and now affordable. It includes the analyses of IS6110 DNA fingerprints, large sequence polymorphisms, spacer oligonucleotide typing (spoligotyping), mycobacterial interspersed repetitive units/variable-number tandem-repeats (MIRU/VNTR), single nucleotide polymorphism (SNP) and more recently, whole-genome sequencing (WGS). Of these, MIRU/VNTR and spoligotyping are the most readily accessible and widely used in developing countries because both have large global reference databases and computational tools that allow assignment of isolates in the major Mtb complex lineages [12, 13, 16–18]. When combined with spatio-temporal data, MIRU/VNTR and spoligotyping are considered the gold standard for identifying and tracking chains of transmission. More importantly, both have become indispensable for TB epidemiology studies of drug resistance within communities and across the world [5, 7, 14, 16, 19, 20]. The optimal TB treatment duration for the different anatomic sites, including cutaneous TB, meninges and pericardial spaces, is unknown [5]. Furthermore, there are very few data on the effect of the standard six-to-nine-month combination therapy on acquired drug resistance and patients’ outcomes for these different anatomic sites constituting EPTB. Here we use spoligotyping to identify TB transmission patterns and to characterize clinical Mtb isolates obtained from EPTB sites in patients treated in the Tshwane metropolitan of South Africa.

The goal of this study was to use agnostic machine learning (ML) algorithms to determine if there are clear patterns among the different anatomic sites impacting drug resistance and/or genotypic clustering of Mtb isolates in affected individuals. Ensembles of ML, such as least absolute selection shrinkage operator, classification regression trees (CART) and random forests, when coupled with stochastic gradient boosting allows identification of weak base learners and detection of nonlinear dependencies in data, including in pharmacometric fields [21–24]. We hypothesized that the majority of EPTB cases will be clustered, with significant proportions bearing drug resistance, which would indicate high rates of transmission of drug resistance in Tshwane. Alternatively, high drug resistance without relationships to clustering would support the ‘pharmacokinetic variability driven *de novo* acquired resistance’ hypotheses [5, 7, 25, 26], where inadequate drug exposures at sites of TB infection lead to selection for drug resistant and drug tolerant strains which eventually leads to therapy failure [20, 21]. Here we used ML to examine patterns that were predictive of drug resistance. We specifically examined whether the different EPTB disease sites, the geographic areas of where the patients came from and period when the patients had disease were also predictive of TB transmission in Tshwane.

## Methods

### Study design

Eligible consecutive clinical samples from EPTB anatomical sites were submitted for diagnosis confirmation by respective DOTS/TB facilities to the National Health Laboratory Services (NHLS). NHLS is the single and integrated laboratory network that covers all public health facilities in South Africa. In Tshwane, NHLS is affiliated with the Department of Medical Microbiology at the University of Pretoria, where microscopy, culture and drug susceptibility testing (DST) of clinical specimens from Tshwane DOTS/TB facilities as well as other nearby provinces, including Limpopo and Mpumalanga, are performed (Fig. 1). All 75 culture positive isolates identified within the six-month study period between July 1st, 2014 and January 31st, 2015 were eligible for enrolment. Isolates that grew nontuberculous mycobacteria or *Mycobacterium bovis* were excluded. Patients’ demographics and clinical data were collected from the specimen request forms. Since the analysed isolates were de-identified and constituted of routinely collected data, the study was not considered human subject research (UP ethical inquiry 143/2015).

### Definition of terms

The case of TB (RVCT) nomenclature and the approach used by the US Centers for Disease Control and Prevention (CDC) was used to assign collected specimen isolates to EPTB anatomic sites for comparison purposes (https://www.cdc.gov/tb/programs/rvct/rvct-form.pdf). According to RVCT, EPTB comprises of pleural, peritoneal, lymphatic, meningeal, genitourinary, laryngeal and unclassified group called ‘other’. We modified the RVCT and replaced laryngeal with cutaneous and then added a ‘disseminated’ category, given the high incidence of those sites in our cohort. Disseminated TB denotes isolates from blood or bone marrow specimens, while patients with a positive isolate from sputum and an EPTB site were grouped separately and denoted ‘PTB/EPTB’. If isolates were obtained from more than one EPTB sites, only the dominant site was recorded. Number needed to screen (NNS) was defined as the number of people that needed to be screened to prevent one TB transmission event or one drug resistance occurrence, based on the assumption that all drug resistance events in the study were acquired during therapy. Isolates that were resistant only to either rifampin or isoniazid were categorized as monoresistant, while those resistant to both were categorized as multidrug resistant TB (MDR-TB).

Spoligotyping examines 43 unique spacer sequences that are interspaced between repetitive sequences in a specific region of difference within the *Mtb* genome. The presence or absence of each of the 43 variable spacers generates strain-specific fingerprints. In this study, isolates were clustered if there was an exact match in all 43 spacers. Cluster name and isolate lineage assignments were made by comparison of fingerprints to international databases: https://www.miru-vntrplus.org/MIRU/index.faces, http://www.pasteur-guadeloupe.fr:8081/SITVIT2/index.jsp [18]. The isolates with unmatched genetic profiles were considered nonclustered or orphan strains. The clustering rate was calculated using the following formula: (nc − c)/n, where nc is the total number of clustered isolates, c is the number of isolate clusters, and n is the total number of isolates. Recent transmission of TB infection was presumed to have occurred when a case had an identical spoligotyping pattern to another case in the cohort during the six-month study period.

### Laboratory testing methods

Isolates identified as M.tb were recovered by subculturing 0.1 ml of the MGIT culture and on Löwenstein-Jansen (LJ) slants to rule out contamination. The slants were placed on their sides and left at room temperature for 24 h and thereafter incubated at 37 °C for 6 weeks. For genomic DNA extraction from Mtb, the isolates were swept off from LJ slants and centrifuged at 10,000 g for 15 min. The supernatant was discarded, and the pellet was re-suspended in 100 ml of sterile distilled water. The isolates were then heat-killed at 95 °C for 20 min in a water bath. This was followed by sonication for 15 min and centrifugation at 13,000 g for 8 min. The supernatant containing-DNA was used for spoligotyping and the Genotype MTBDR*plus* line-probe assay (Hain LifeSciences, Nehren, Germany) while the remainder was stored at − 20 °C.

The Genotype MTBDR*plus* v2 assay was performed to search for mutations associated with rifampin and isoniazid resistance according to the manufacturer’s instructions. Briefly, PCR (50 μL/tube; 40-cycle program) was performed using the HotStar *Taq* DNA Polymerase (Qiagen, Cambridge, MA, United States of America). The PCR products were hybridized following the manufacturer’s instructions. After hybridization, membrane strips were attached to the evaluation sheet, read, and interpreted manually. Spoligotyping was performed as previously described by Kamerbeek and colleagues [27]. The results were analysed using the BioNumerics Software ver. 7.5 (Applied Maths, Kortrijk, Belgium). We assigned each study isolate spoligotype pattern a Spoligotype International Type (SIT) number using the most current international spoligotyping databases comprising of 111,635 clinical isolates [28].

### Classification and regression tree (CART) modelling with stochastic gradient boosting

Stochastic gradient boosting was used to identify weak base learners, determine meaningful pairwise interactions and the percent of variance attributable to those interactions, variable important scores for those variables and identify thresholds for decision-making [29–33]. Important variables define the most influential predictors, including both linear and nonlinear rules that appear in the predictive model [33]. We used the methods of Leo Breiman [34], a pioneer in ML algorithms and artificial intelligence, and Jerome Friedman [30]. Multivariate adaptive regression spline (MARS) models for binary targets in classification problems implemented in TreeNet version 8.3 software were also used for graphic visualization. Details of the modelling approaches that use these ML algorithms and tools for pharmacokinetics and pharmacodynamics (PK/PD) analyses, pharmacometrics and for general decision-making purposes in the clinic has been published before and reviewed within [21–23, 25, 35, 36]. Optimal CART for drug resistance and clustering outcomes were also graphically depicted for illustrative purposes. The variable importance scores from Random forest were used to rank and identify variables most predictive of acquired drug resistance or clustering. CART and MARS in TreeNet were used to identify thresholds for continuous variables applied to clinical decision-making, as we have done in the past [21]. Similarly, both algorithms were used to group categorical variable that were considered similar, based on GINI criteria. Five-fold cross validation was used with all models which included all patients’ clinical characteristics shown in Table 1 and Mtb isolates’ spoligotypes. Area under the receiver operating characteristic curve, misclassification rates and the F1-statistics were used for model comparison. Parsimony was also used to select the final models.

**Table 1:** Demographic and clinical characteristics of all patients

Variable		ALL	Lineage of *Mycobacterium tuberculosis* isolate				*P*-value
	Level	*N* = 70 (%)	Orphan, *n* = 12 (%)	2 (East Asian); *n* = 25 (%)	3 (East-African Indian); *n* = 6 (%)	4 (Euro-American); *n* = 27 (%)	
Demographic							
Sex	Female	28 (40%)	5 (42%)	5 (20%)	5 (83%)	13 (48%)	*0.021*
	Male	42 (60%)	7 (58%)	20 (80%)	1 (17%)	14 (52%)	
Age	Median (range); years	34 (1–82)	33.5 (12–61)	36 (1–60)	38.5 (32–82)	33 (9–50)	0.492
Age groups	Pediatric (<16y)	5 (7%)	1 (8%)	3 (12%)	0	1 (4%)	0.684
	Adult (>16y)	65 (93%)	11 (92%)	22 (88%)	6 (100%)	26 (96%)	
TB/DOTS Facilities	Folang	1 (1%)	0	1 (4%)	0	0	0.328
	Kalafong	26 (37%)	4 (33%)	8 (32%)	5 (83%)	9 (33%)	
	Mamelodi	10 (14%)	1 (8%)	2 (8%)	1 (17%)	6 (22%)	
	Potchefstroom	4 (6%)	0	0	0	4 (15%)	
	Pretoria West	4 (6%)	1 (8%)	2 (8%)	0	1 (4%)	
	Skinner	2 (3%)	1 (8%)	0	0	1 (4%)	
	Steve Biko	14 (20%)	3 (33%)	6 (24%)	0	4 (15%)	
	Tshwane	9 (13%)	1 (8%)	6 (24%)	0	2 (7.41%	
Clinical							
Disease site	Pleural effusion	20 (29)	2 (17)	4 (16)	3 (50)	11 (41)	0.489
	Lymphadenitis	20 (29)	4 (33)	11 (44)	1 (17)	4 (15)	
	Cutaneous TB	10 (14)	3 (25)	1 (4)	1 (17)	5 (19)	
	Peritoneal effusion	5 (7)	0	3 (12)	0	2 (7)	
	Meningeal TB	5 (7)	1 (8)	2 (8)	1 (17)	1 (4)	
	Disseminated	4 (6)	1 (8)	2 (8)	0	1 (4)	
	Genitourinary	2 (3)	0	0	0	2 (7)	
	Other	4 (6)	1 (8)	2 (8)	0	1 (4)	
DR	RIF/INH susceptible	59 (84)	9 (75)	22 (88)	5 (83)	23 (85)	0.125
	RIF monoresistant	6 (9)	3 (25)	0	0	3 (11)	
	INH monoresistant	3 (4)	0	2 (8)	0	1 (4)	
	MDR-TB	2 (3)	0	1 (4)	1 (17)	0	

*RIF* Rifampin; *INH* Isoniazid; *MDR-TB* Multidrug resistant tuberculosis; *DR* Drug resistance

### Statistical analysis

Output from the gradient boosting ML were used to calculate attributable risk (AR), NNS and in multivariate logistic regression models. Newcombe/Wilson scores with continuity correction were used in computing AR 95% confidence intervals (CI) [37], otherwise exact binomial methods of Klopper-Pearson were employed. The STATA (College Station, Texas) and GraphPad software (San Diego, California) were used for statistical analysis. Fisher’s exact test was used to compare proportions, while the Kruskal–Wallis test compared median values between groups. All tests were two-sided and set at an alpha of 5%.

## Results

**Fig. 2:**
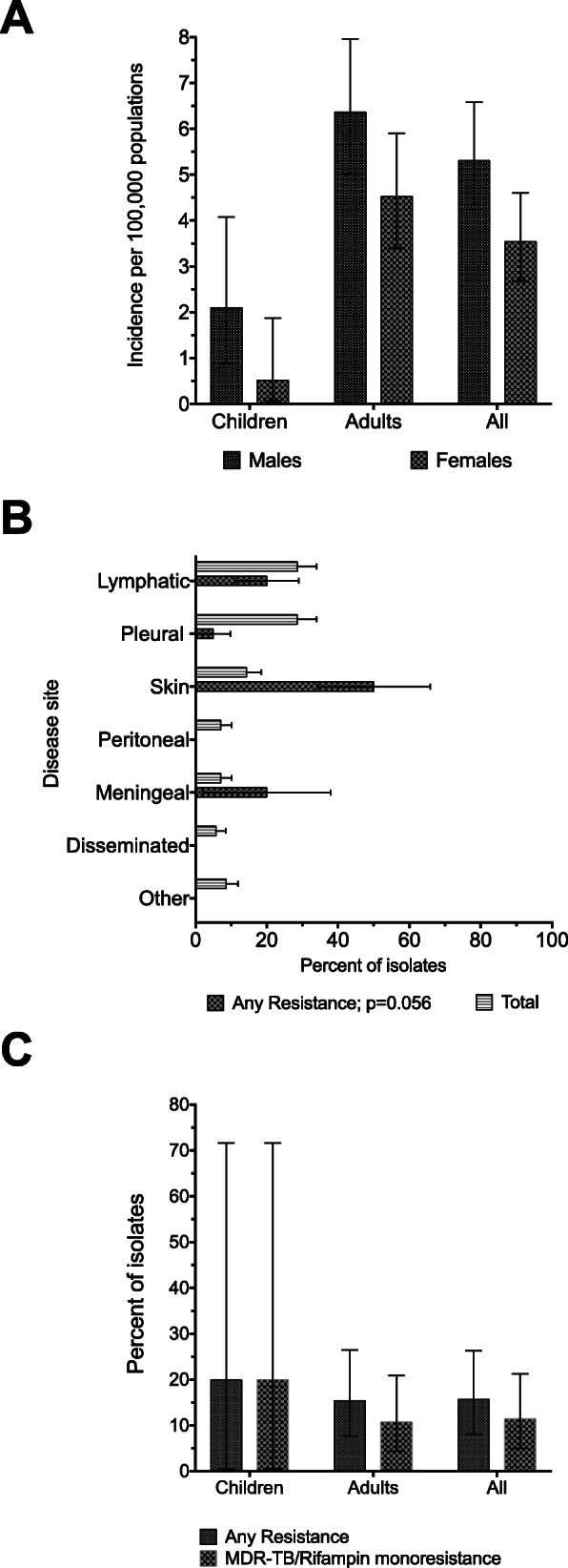
Population estimates of extrapulmonary tuberculosis (EPTB) and proportion with drug resistance in Tshwane by age group and sex in 2015. Figure 2**a** show that the estimated EPTB incidence stratified by age and gender. As shown the estimates in females was 3.54 (95% CI: 2.68–4.60), while that in males was 5.31 (95% CI: 4.24–6.58) per 100, 000 populations for the year 2015. Figure 2**b** show the proportion of total isolates (*N* = 70) by anatomic EPTB and within each category the percent of isolates with resistance to either rifampin or isoniazid or both. As shown, none of the isolates from peritoneal specimen, disseminated (i.e., blood or bone marrow) and other specimen samples were drug resistant. Figure 2**c** stratifies drug resistance by age, and as shown one out of the 5 isolates from children were drug resistant, and that same isolate was also rifampin resistance

Of the 75 unique and consecutive isolates submitted, from eight out of the 25 DOTS/TB facilities in Tshwane we excluded from further analysis five (7%) isolates because they grew *Mycobacterium bovis*. All excluded isolates were from children < 16 years. Of the remaining 70 (93%) isolates, there were 28 females (40%). The overall proven EPTB incidence was 4.43 (95% CI: 3.72–5.23) per 100,000 population per year in Tshwane (Fig. 2a). Even though the ages varied widely from 1 year to 85 years, only five (7.14%) samples were from children < 16 years (Table 1). Detailed demographic, clinical and genotyping data in Table 1 show that women were significantly overrepresented among patients with the East-Africa-Indian genotypes or lineage 3. Figure 2b shows that the most frequently encountered proven EPTB disease sites were pleural and lymphatic, each accounting for 29% (95% CI: 18–41%), and cutaneous TB which accounted for 14% (95% CI: 7–25%), while peritoneal and meningeal TB each accounted for only 7% (95% CI: 2–16%) of the cases. The associations between major Mtb lineages, EPTB disease sites or DOTS/TB facilities were not statistically significant.

### *Mycobacterium tuberculosis* spoligopatterns clustering and inferred transmission

**Fig. 3:**
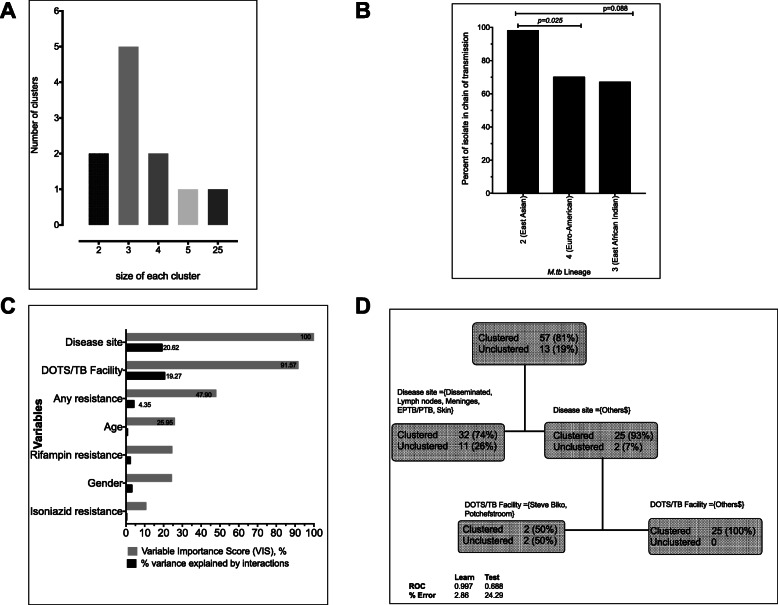
Clustering and chains of *Mycobacterium tuberculosis* transmission. The number of clusters and the sizes of each cluster are shown in Fig. 3**a**, while the proportion of patients from each the major genotype lineages (2, 3 and 4) in a chain of transmission are depicted in Fig. 3**b** (there were no isolates from lineage 1 enrolled in study). Variable importance scores and proportion of the variance explained by interactions between variables were obtained from stochastic gradient modeling of between 200 and 2000 classification and regression trees (CART) are shown in Fig. 3**c**, while the optimal and sample tree from those models is shown in Fig. 3**d**. Disease site was the most important variable at the apex with 100%, while DOTS/TB Facility was second with 92% relative to disease site. However, between variables interactions explained 21% of the variance for disease site and 19% for DOTS/TB Facility (Fig. 2c) which means that there are important nonlinear interactions accounting clustering variance. Figure 3**d** shows disease site and DOTS/TB Facility interactions significantly influence clustering, even though each individual variable was not statistically significant in Table 2 based on Fischer’s exact test. As shown in, isolates from disseminated diseases, lymph nodes, meninges, EPTB/PTB and skin were significantly less to be clustered; 32/43 (74%) versus 25/27 (93%), when compared to the rest of disease site. The receiver operating characteristics curve. (ROC) for this single node is 0.744 (95% confidence interval [CI] 0.590–0.991). The model is reproducible as demonstrated by the test ROC of 0.688 and error rate of < 3% on the training model

The majority of Mtb isolates, 57/70 (81%), occurred in clusters that varied in size from two to 25 isolates. The largest cluster comprised 25 (36%) isolates belonging to the Beijing clade, an East Asian lineage also called lineage 2 (Fig. 3a). Mtb isolates from the three major lineages 2, 3 and 4 were in chain of transmission for 98, 67 and 70% of the isolates, respectively (Fig. 3b). Thus, the Beijing strains were significantly more likely to occur within a chain of EPTB transmission when compared a Euro-American strain: odds ratio (OR) = 10.11 (95% CI 1.56–116). On the other hand, 12/13 (92%) of unclustered isolates were orphans in the international spoligotyping databases, while the remaining isolate belonged to the X2 clade, which is of the Euro-American lineage. Table 2 shows that there was no significant association between clustering and the variety of demographic and clinical factors, including notably drug resistance and DOTS/TB facilities, in bivariate analyses based on standard statistical tests.

**Table 2:** Association between demographic and clinical factors with M. tuberculosis genotypes clustering

		Clustering			Univariate
**Variable**	Level	Unclustered, *n* = 13 (%)	Clustered, *n* = 57 (%)	*P*-value	Odds ratio (95% CI)
*Demographics*					
**Sex**	Female	6 (46%)	22 (39%)	0.789	Referent
	Male	7 (54%)	35 (61%)		1.36 (0.41–4.49)
**Age groups**	Adults (> 17 y)	12 (92%)	53 (93%)	1.000	Referent
	Children = < 16 y	1 (8%)	4 (7%)		0.91 (0.09–8.85)
**TB/DOTS Facilities**	Folang	0	1 (2%)	0.735	1--
	Kalafong	5 (38%)	21 (37%)		0.53 (0.05–5.21)
	Mamelodi	1 (8%)	9 (7%)		1.13 (0.06–21.08)
	Potchefstroom	0	4 (7%)		1--
	Pretoria West	1 (8%)	3 (5%)		0.38 (0.02–8.10)
	Skinner	1 (8%)	1 (2%)		0.13 (0–4.00)
	Steve Biko	4 (31%)	10 (18%)		0.31 (0.03–3.38)
	Tshwane	1 (8%)	8 (14%)		Referent
*Clinicals*					
**Any drug resistance**	Susceptible	10 (77%)	49 (86%)	0.416	Referent
	Resistance	3 (23%)	8 (14%)		0.54 (0.12–2.42)
**INH mono-resistance**	Susceptible	13 (100%)	52 (91%)	0.576	1--
	Resistance	0	5 (9%)		
MDR/RIF*	Susceptible	10 (77%)	49 (91%)	0.179	Referent
	Resistance	3 (23%)	5 (9%)		0.34 (0.07–1.66)
**Disease site**	Disseminated	1 (8%)	3 (5%)	0.540	Referent
	Lymph nodes	4 (31%)	16 (28%)		1.33 (0.11–16.48)
	Meninges	1 (8%)	4 (7%)		1.33 (0.06–31.12)
	EPTB/PTB	1 (8%)	2 (4%)		0.67 (0.02–18.06)
	Pericardium	0	1 (2%)		1--
	Peritoneum	0	5 (9%)		1--
	Pleura	2 (15%)	18 (32%)		3 (0.20–44.36)
	Genitourinary	0	2 (4%)		1--
	Cutaneous TB	4 (31%)	6 (11%)		0.5 (0.04–6.68)
**Beijing Clade**	Yes	0	25 (44%)	***0.003***	--1
	No	13 (100%)	32 (56%)		
**Euro-American**	No	12 (92%)	31 (54%)	***0.011***	
	Yes	1 (8%)	26 (46%)		10.06 (1.23–82.64)
**East-Africa-India**	No	13 (100)	51 (89)	0.221	
	Yes	0	6 (11%)		--1

*MDR-TB* Multidrug resistant tuberculosis; *RIF* Rifampin; *INH* Isoniazid; *EPTB* Extra-pulmonary TB

Next, we applied stochastic gradient ML algorithms to identify the important variables that predicted clustering and to determine if there were nonlinear associations that could explain genotypic clustering patterns (Fig. 3c-d). The results shown in Fig. 3c revealed that specific EPTB disease site and DOTS/TB facilities as well as any drug resistance were ranked important variables and that nonlinear interaction between these accounted for almost 45% of clustering variance. The usual factors described in TB epidemiology, such as age, were either less prominent or scored zero (in the case of patients’ gender). The pooled isolates from disseminated TB, lymphadenitis, meningitis, EPTB/PTB and cutaneous TB disease site were less likely to be clustered compared to those not from the same TB disease sites: OR = 0.23 (95% CI: 0.10–0.99) and the attributable risk (AR) was 0.18 (95% CI: 0.01–0.40). The OR and AR for clustering improved to 0 (95% CI: 0–0.45) and 0.26 (95% CI: 0.10–0.47), respectively, if DOTS/TB facilities were also used in the combination screening (Fig. 3d). If these two factors are used as screening tools the overall NNS to prevent transmission of one TB case would be 3.91 (95% CI: 2.14–10.32). The sensitivity for the CART shown in Fig. 3d was 0.56 (95% CI: 0.43–0.68), while the positive predictive value was 0.74 (95% CI: 0.60–0.85). However, both the specificity and negative predictive values were poor. Nonetheless, when combined, these data show that disease site and DOTS/TB facilities, i.e., geographic information systems, can be used in combination with isolates genotypes to identify situations where TB transmission is taking place, even for paucibacillary forms of the diseases such as EPTB.

### Predictors of EPTB drug resistance

The majority, 59/70 (84%), of the isolates were susceptible to both rifampin and isoniazid, while two (3%) isolates were MDR. However, rifampin resistance was observed in disproportionately large proportions of isolates, 8/70 (11%), which is rather unusual, since isoniazid monoresistance was observed in only 3/70 (4%) isolates (Fig. 2c). Nonetheless, Table 3 shows that there was no association between drug resistance and most demographic and clinical factors examined, including clustering (*p* = 0.419) or Mtb spoligotypes (*p* = 0.737) for any resistance, based on straightforward frequentist tests. The exception was between rifampin resistance and the disease site: *p = 0.036*.

**Table 3:** Association between demographic and clinical factors with drug resistance

		Any resistance			Rifampin, MDR-TB		
**Variable**	Level	Pan-susceptible, *n* = 59 (%)	Resistant, *n* = 11 (%)	*P-*value	Pan-susceptible, *n* = 59 (%)	Resistant, *n* = 8 (%)	*P*-value
*Demographic*							
**Sex**	Female	24 (41%)	4 (36%)	0.789	24 (41%)	2 (25%)	0.393
	Male	35 (59%)	7 (64%)		35 (59%)	6 (75%)	
**Age groups**	Adults (> 17 y)	55 (93%)	10 (91%)	0.785	55 (93%)	7 (88%)	0.563
	Children = < 16 y	4 (7%)	1 (9%)		4 (7%)	1 (12%)	
**TB/DOTS Facilities**	Folang	1 (2%)	0	0.958	1 (2%)	0	0.856
	Kalafong	21 (36%)	5 (45%)		21 (36%)	4 (50%)	
	Mamelodi	9 (15%)	1 (9%)		9 (15%)	0	
	Potchefstroom	4 (7%)	0		4 (7%)	0	
	Pretoria West	3 (5%)	1 (9%)		3 (5%)	1 (12%)	
	Skinner	2 (3%)	0		2 (3%)	0	
	Steve Biko	11 (19%)	3 (27%)		11 (19%)	2 (50)	
	Tshwane District Hosp.	8 (14%)	1 (9%)		8 (14%)	1 (13%)	
*Clinical*							
**Genotypes**	Beijing	22 (37%)	3 (27%)	0.737	22 (37%)	1 (12%)	0.350
	Cas_KILI	4 (7%)	0		4 (7%)	0	
	EAI1_SOM	1 (2%)	1 (9%)		1 (2%)	1 (12%)	
	LAM11_ZWE	2 (3%)	0		2 (3%)	0	
	LAM3	4 (7%)	0		4 (7%)	0	
	LAM4	2 (3%)	1 (9%)		2 (3%)	1 (12%)	
	LAM9	2 (3%)	1 (9%)		2 (3%)	1 (12%)	
	S	3 (5%)	0		3 (5%)	0	
	T1	3 (5%)	0		3 (5%)	0	
	X1	4 (7%)	1 (9%)		4 (7%)	0	
	X3	2 (3%)	1 (9%)		2 (3%)	1 (12%)	
**Unclustered**	Orphan/X2	10 (17%	3 (27%)	0.419	10 (17%)	3 (38%)	0.168
**Disease Site**	Disseminated	4 (7%)	0	0.134	4 (7%)	0	***0.036***
	Lymph nodes	16 (27%)	4 (36%)		16 (27%)	4 (38%)	
	Meninges	4 (7%)	1 (9%)		4 (7%)	0	
	EPTB/PTB	3 (5%)	0		3 (5%)	0	
	Pericardium	1 (2%)	0		1 (2%)	0	
	Peritoneum	5 (8%)	0		5 (8%)	0	
	Pleura	19 (32%)	1 (9%)		19 (32%)	0	
	Genitourinary	2 (3%)	0		2 (3%)	0	
	Cutaneous TB	5 (8%)	5 (45%)		5 (8%)	5 (63%)	

*MDR-TB* Multidrug resistant tuberculosis; *EPTB* Extra-pulmonary TB; *PTB* Pulmonary TB

**Fig. 4:**
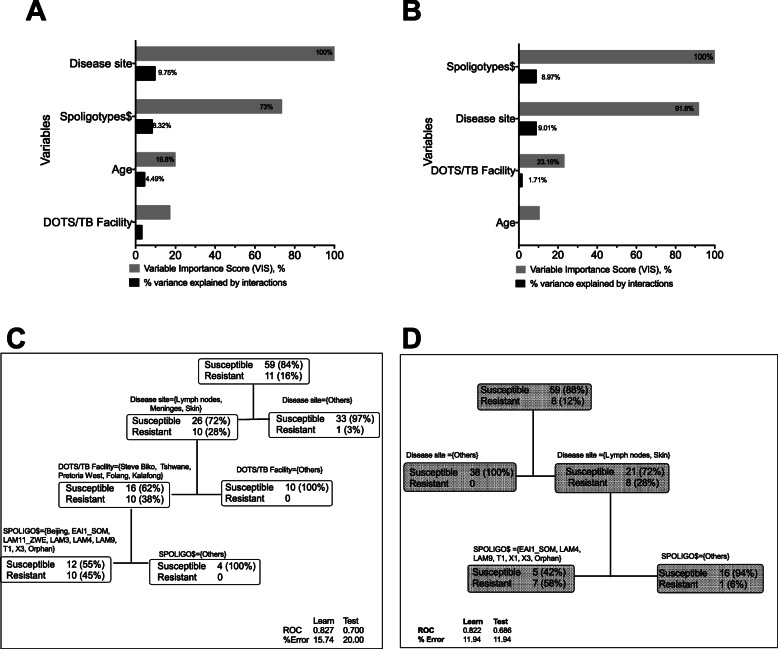
Predictors of drug resistance in *Mycobacterium tuberculosis* isolates from extra-pulmonary sites. The variable importance scores and proportion of the variance explained by interactions between the variables that were obtained from stochastic gradient modeling for any drug resistance are shown in Fig. 4**a**, while those for MDR-TB/Rifampin monoresistance are shown in Fig. 4**b**. Multivariate adaptive regression trees (MARS) for binary outcomes with two-way interactions detection were made in the TreeNet software. The optimal representative classification and regression trees (CART) are shown in Fig. 4**c** for any resistance and in Fig. 4**d** for MDR-TB/Rifampin monoresistance. The primary node (disease site) for any drug in Fig. 4**c** is almost identical to that for MDR-TB/Rifampin monoresistance in Fig. 4**d**, the difference being addition of meninges to the former group. The sensitivity for both is 0.72 (95% CI: 0.56–0.84). However, positive predictive value for the former is 0.44 (95% 0.32–0.57) and for the latter is 0.36 (95% 0.25–0.48). The MDR-TB/Rifampin monoresistance group necessarily excludes the three isoniazid monoresistance isolates, hence the overall number of isolates analyzed in Figs. 4**c/d** are 67 and not 70

ML analyses revealed the differential impact of the interactions between disease site and Mtb genotypes on any drug resistance and especially MDR-TB/rifampin monoresistance (Fig. 4a/b). Firstly, disease sites characterized by sanctuary states, i.e., lymphadenitis, meningitis and cutaneous TB, were significantly more likely to be associated with any drug resistance: OR = 12.69 (95% CI: 1.82–141.60) and AR = 0.25 (95% CI: 0.06–0.43), when compared to EPTB in other sites. Secondly, with regards to MDR-TB and/or rifampin monoresistance, the top predictor was lymphadenitis and cutaneous TB disease, which means that meningitis was excluded. This is not surprising since rifampin does not penetrate well into the blood-brain barrier and the current doses given are so low that virtually none gets into the cerebrospinal compartment. The sensitivity and specificity of using disease site as proxy to identify isolates likely to be MDR-TB or rifampin monoresistant are 1.00 (95% CI: 0.68–1.00) and 0.64 (95% CI: 0.52–0.75), respectively. When information about likely Mtb genotypes is added, as shown in Fig. 4d, the specificity improves to 0.84 (95% CI: 0.71–0.92). These data show that for every four patients (95% CI: 2.11–10.64) with TB lymphadenitis or cutaneous TB, we would expect one or more to have MDR-TB and/or rifampin monoresistance when compared to those with TB in other sites. This means that screening patients using drug susceptibility tests and changing the treatment regimens would prevent therapy failure and reduce transmission of drug resistant TB.

## Discussion

This study focused on characterizing clinical Mtb isolates in real-world settings and hence has limitations compared to observational studies, including inadequate sample size, imprecise and some missing information (e.g. HIV infection status). First, we only used spoligotypes to assign clusters, which limits and biases the clustering resolution and might potentially over-estimate clustering rates. The second limitation relates to a small sample size and misclassification of EPTB disease sites, which has notoriously confounded comparison of EPTB incidences between studies [38]. Previous studies have identified disease site-specific risk factors, including those with certain Mtb genotypes, drug resistance and meningeal TB which we could not reproduce in our study, since only five (7%) meningeal TB and 1 (1%) pericardial TB isolates were enrolled [39–43]. Nonetheless, we used validated RVCT methods to allow comparisons between studies. Third, the Mtb isolates were not serialized, and information on drug therapy received, TB drug doses and period of isolates’ collection relative to TB therapy, was not available. This made inference and distinction of primary transmitted resistance from acquired resistance in our study difficult [44]. Incomplete medical history on the laboratory request form also made it difficult to determine which isolates were from patients immunosuppressed with HIV or concomitant immunosuppressive agents used for rheumatological diseases. Nonetheless, with ML modelling which is more suitable with missing data or highly complex data structure, we were able to demonstrate that routinely collected laboratory and clinical data can be used to screen patients and identify risk groups where acquired drug resistance is most likely to occur. Gradient boosting allows identification of weak predictors, nonlinear relationships and thresholds in the data space [32], which is like the proverbial “finding a needle in a haystack”. In this case one uses giant magnets to find that needle. Sensitivities and specificities > 84% are reasonable and acceptable, given that the information required for initial screening (i.e., identifying disease site as lymphadenitis, cutaneous TB or meningitis) can be ascertained by history and clinical examination. Moreover, ROC values ~ 70% with cross validation somehow reassures us that results such as these are likely to be reproducible with similar populations. Unlike most EPTB studies performed at large specialized hospitals [45, 46], our study has minimal referral bias, hence the other strength of this study is that the isolates were from primary DOTS/TB facilities and not from patients treated at tertiary specialized facilities.

There are three notable findings from our study with important public health policy and TB control efforts that target reduction of both disease transmission and drug resistance. The findings are certainly applicable in the Tshwane metropolitan area and have potential relevance across similar urban populations in South Africa and across the sub-Sahara African metropolis. The first key finding is the hierarchical and nonlinear association between key EPTB disease sites (mainly lymphatic, cutaneous TB and meningeal) and spoligotypes (mainly to impact both disease transmission and drug resistance). The association between Beijing strains and both TB disease transmission and drug resistance has been well described in South Africa and across the world and the results have been mixed [9–11, 15, 47–49]. Our study demonstrates that those relationships are conditional, complex and characterized by several nonlinear interactions (**Figs.** **3** and **4**). For example, two-way interactions between EPTB disease site and another variable explained > 20% of the variance in clustering and almost 10% of drug resistance. This means that unless those nonlinear relationships are fully examined, the purported factors driving either transmission or drug resistance will be highly biased or wrong. In fact, for both clustering and drug resistance, the impact of Mtb genotypes is of second order, suggesting that some mycobacterial genotypes have increased propensity to act on some EPTB disease sites and less likely on others. The differential impact of EPTB disease site on any drug resistance (shown in Fig. 4c) and on MDR-TB/rifampin monoresistance (shown in Fig. 4d), is revealing and consistent with standard PK/PD principles underlying drug resistance emergence [25, 50–52]. PK variability between individuals mean that some patients will have faster drug clearances than others when given the same drug dose. Therefore, inadequate drug exposures at the site of infection, which occurs because of PK variability or suboptimal drug doses or poor drug penetration into protected sites such as meningeal or pericardial spaces, leads to selection of drug resistant or drug tolerant isolates. The selected mutants eventually acquire putative mutations in time. In other words, acquired drug resistance (ADR) occurs de novo during therapy primarily because of inadequate dosing or with unoptimized therapy regimens. The WHO recommends the same standardized and uniform therapy regimens and doses used for PTB for EPTB, with the caveat of experts’ opinions that varying longer therapy durations be given for meningeal and bone/joints disease sites [53]. Indeed, these same guidelines are used in Tshwane. As shown in Figs. 4c-d, the standard WHO recommended EPTB treatment regimen is associated with drug resistance in certain EPTB sites such as lymphatic, cutaneous TB and meningeal site. In this study, the attributable risk for both any resistance and MDR-TB and/or rifampin monoresistance were substantial: 0.25 and 0.64, respectively. The corollary suggestion from this specific finding is that the majority resistance observed in our study is more likely acquired during therapy rather than being ‘pre-existing’ or primary. The NNS for targeted screening among EPTB patients based on disease sites for any resistance was 4 and for MDR-TB and/or rifampin monoresistance was 2, which is even more efficient and effective than widely recommended population screenings for active TB in congregate settings or among select risk groups, such as patients with diabetes mellitus or HIV [54]. For example, the NNS HIV infected patients to find one active TB case in regions with low TB incidences is 25 (ranges 11–144) and in high TB incidence regions is 10 (ranges 5–22), while that for prisoners is 520 (ranges 69–427) and 43 (ranges 21–123), respectively.

Secondly, even though the proportion of EPTB disease sites were similar to previous observations, the overall incidence of proven EPTB of 4.43 per 100,000 populations was lower than expected. There were 8034 microbiologically confirmed PTB cases in Tshwane in 2015, an estimated incidence rate of 254 (95% CI: 249–260) cases per 100,000 population [1]. Confirmed PTB status was based on positive GeneXpert MTB/RIF assay, cultures, line probe assays and microscopy smears, which probably overestimated confirmed PTB cases by accepting nontuberculous mycobacteria cases, based microscopy smears. Hoogendoorn et al. reviewed charts of patients treated and notified for clinical EPTB in the predominantly rural Limpopo province, for 10 months of 2013 [3]. Of the 336 patients diagnosed, only 57% had good evidence for stated diagnoses. Nonetheless, the overall estimated incidence of clinical EPTB in that study was 27.92 (95% CI: 24.80–31.23) and that for clinical meningeal TB was 2.56 (1.70–3.70) per 100,000 populations per year. Meningeal TB comprised 9.82% (95% CI: 6.86–13.52) of EPTB in Limpopo and 9.04% (95% CI: 6.94–11.54) in Soweto, per year [3, 46]. Our estimates of EPTB incidence in Tshwane is six-fold lower than those reported from Limpopo; however, proven meningeal TB comprised 7.14% (2.36–15.89) of cases in Tshwane, suggesting that the meningeal TB proportions were similar between these disparate South African studies. In the US, EPTB as a proportion of total TB cases has been steadily increasing as TB elimination efforts are accelerated and the WHO TB elimination targets getting realized. From 7.6% in 1962 at the peak of the epidemic when TB incidence was 28.6 per 100, 000 population, EPTB increased to 15.7% in 1993 with the HIV resurgence and was 30.9% % in 2017 when the reported TB was 2.8 per 100, 000 population [55]. Contrary to the explanations given by Hoogendoorn and others, we actually hypothesize that with the widespread use of laboratory methods to prove EPTB, the incidence will increase consistent with observations in the US, where majority of EPTB reported are proven TB. We argue that several cases currently reported as clinical EPTB by Hoogendoorn and others in South Africa and elsewhere in low-resources settings could be due to other bacterial infections or due to systemic inflammation from HIV infection.

EPTB is generally paucibacillary in nature which means that usually there are not enough Mtb bacilli in tissues from which cultures can be obtained; histology samples are not easy to obtain and therefore not routinely collected. Culture positivity and histology examination of clinical samples, which are the gold standards for confirming EPTB, are notoriously low (about 15% in high TB burden areas) and inconsistent when compared against clinically suspected TB cases. Investments in improved diagnostics to confirm EPTB or ML algorithms that are trained on large clinical data to predict EPTB, will not only save lives by reducing unnecessary TB treatments, but will also be cost-effective because of the reduction of TB transmission and ADR. Both interventions will accelerate meeting WHO TB elimination targets.

Finally, with regards to the heterogeneity of the Mtb spoligotypes causing EPTB, the general predominance of the Beijing clades (lineage 2) and the Euro-American lineage 4 within the Tshwane metropolis are in concordance with the work of others [19]. This is not surprising since lineages 2 and 4 are thought to be the most successful strains among all the Mtb complex organisms in causing all forms of TB disease, including PTB [13, 18, 20]. Previous reports have associated Mtb lineages of Beijing clade with major outbreaks in different parts of the world and was shown to disseminate more rapidly and caused more-severe disease than other strains [21–23]. Moreover, several other epidemiological data suggest that certain *Mtb* genotypes, such as the W-Beijing genotypes, are more transmissible than others [20–22]. Our study found that the Beijing strains within a chain of EPTB transmission was statistically significant when compared to the Euro-American and East African Indian strains which might support the variable virulence hypotheses [23, 24].

## Conclusion

The majority of Mtb strains circulating in the city of Tshwane metropolis belong to the East Asian (predominantly Beijing clade), Euro-American and East-African Indian lineages. Each of these are likely to be clustered, suggesting on-going transmission of both drug-susceptible and drug-resistant EPTB. However, the proportion attributed to transmission was significantly higher with the East Asian lineage compared to the other lineages, which might support the variable virulence hypothesis. On the other hand, the proportion of drug resistance, especially rifampin resistance, attributable to certain sanctuary EPTB sites, including lymph nodes, meninges and cutaneous TB, was significantly higher, 25% (95% CI: 6–43%), when compared with other EPTB sites. This observation suggests that low rifampin exposures, due to poor penetration into those sites or inadequate rifampin doses, significantly contribute to ADR, which is also consistent with PK/PD principles of pharmacokinetics variability. Moreover, the significant nonlinear relationship between EPTB sites, Mtb genotypes and drug resistance (particularly MDR-TB and/or rifampin monoresistance) observed, is consistent with prior clinical observations. Together, these data suggest that inadequately treated EPTB is contributing to drug resistance and overall poorer outcomes.

## Data Availability

The original data and materials from this study is stored in the National Health Laboratory Track Care laboratory data base in South Africa. Analyzed copy of data are available from the corresponding author on reasonable request.

## Abbreviations

TB: Tuberculosis
PTB: Pulmonary tuberculosis
EPTB: Extra-pulmonary tuberculosis
DST: Drug susceptibility testing
LPA: Line probe assay
INH: Isoniazid
MDR-TB: Multidrug resistant tuberculosis
RIF: Rifampin
WHO: World health organization
NHLS/TAD: National health laboratory services/tshwane academic division
MRC: Medical research council
